# An evaluation of access to critical breast oncoplastic patient information within a regional autologous free-flap reconstruction service

**DOI:** 10.3332/ecancer.2019.ed92

**Published:** 2019-07-18

**Authors:** Muneer Ahmed, Simon Wood, Daniel Leff

**Affiliations:** 1 Department of Breast Surgery, Charing Cross Hospital, Fulham Palace Road, London W6 8TF, UK; 2 Department of Plastic Surgery, Charing Cross Hospital, Fulham Palace Road, London W6 8TF, UK; 3 Department of Biosurgery and Surgical Technology, Imperial College London, St Mary's Hospital, Praed Street, Paddington, London W2 1NY, UK

**Keywords:** breast neoplasms, mastectomy, free tissue flaps, information technology, clinical governance, patient safety, workload, tertiary care centers

## Abstract

The configuration of autologous free-flap breast reconstruction services within regional hubs means that increasing numbers of patients operated upon at these units will be external referrals without their oncological investigations carried out at the tertiary receiving centre. Oncoplastic guidelines exist that clearly state the minimum necessary patient information that should be provided by the referring unit to the reconstruction centre. However, the logistics of such critical information transfer can be challenging, but it is essential that such issues are addressed to ensure safe oncological management of patients undergoing autologous breast reconstruction.

The rising incidence of breast cancer, combined with improved cancer survivorship, genetic assessment and patient awareness has placed increasing demand upon needs for breast reconstruction. Guidelines recommend that patients should be offered a range of reconstruction options during their cancer pathway—including those not necessarily available at their primary treating facility [1]. However, within the United Kingdom (UK) only half of National Health Service (NHS) Trusts employ a consultant plastic surgeon—compared to almost all employing a consultant breast surgeon [2]. This has meant that complex autologous-tissue based reconstruction has had to be centralized to regional hubs, which inevitably have a high caseload. Whilst this approach allows complex reconstruction to be accessed by a greater proportion of women, it does raise several logistical issues with particular reference to surgical oncological management. The Association of Breast Surgeons (ABS) and British Association of Plastic and Reconstructive Surgeons (BAPRAS) Oncoplastic Guidelines [3] state that, where patients are referred to a different unit for their care, all information should be made available to the receiving unit. This information includes documentation of the multidisciplinary team (MDT) meeting outcome prior to referral; imaging, cytology, pathology and other relevant reports; all imaging and histopathology slides for review; details of co-morbidity, psychological, psychiatric and other relevant medical history [3]. This information is considered the minimum critical information required by a receiving reconstruction unit to safely function.

However, transfer of such standard, essential data is not without its own difficulties. We prospectively evaluated these potential logistical issues within our complex autologous reconstruction unit—serving a population of 2 million people in North-West London—which receives external referrals from across this region. Over a 6-month period from October 2018 to April 2019 all patients undergoing autologous breast reconstruction (internal and external referrals)—as recorded on a prospectively maintained database—were evaluated for the operating surgeon availability of the gold-standard ABS/BAPRAS Oncoplastic Guideline dataset on the Trust’s online information system (CERNER^TM^) on the day of reconstructive surgery. Of 61 patients undergoing free-flap reconstruction, just over half (31/61) were external referrals to the tertiary referral centre (Charing Cross Hospital). Of these 31 external referrals, only 9 patients had their oncological surgery—skin-sparing mastectomy and axillary staging—performed by the surgical oncologist from the referring centre. Sixteen patients had not been clinically assessed at Charing Cross Hospital Breast Clinic and 5 had no recorded MDT discussion. For 16 patients, a formal histopathological report confirming their breast cancer diagnosis had to be obtained on the morning of surgery. Thirty-four patients underwent upfront sentinel node biopsy or fine-needle aspiration (FNA) of abnormal axillary nodes with results only obtained on the day of mastectomy and reconstruction in just under half (14/34) of these patients. Mammography and ultrasonography imaging were not available for review in 23 patients and of 31 patients who underwent magnetic resonance imaging (MRI), 27 did not have formal imaging available for review. For the 31 external referrals, the median email correspondence received by the Charing Cross Hospital breast MDT co-ordinator for managing care was 4 emails with enclosed documentation per patient (range 1-11).

This snapshot of difficulties in accessing gold-standard information in a regional tertiary referral reconstruction unit is unlikely to be in isolation and applicable to anywhere with centralization of services. Interestingly, during this evaluation period, no serious adverse events were recorded. This demonstrates that despite the absence of easily accessible data online, the operating surgeon was forced to access it via different means to perform the surgical oncological component of the reconstruction. Clearly, this is time-consuming, labour-intensive and risks delay, or cancellation of scheduled surgery involving multiple-teams should the data not become available.

There are different potential approaches to tackling these issues. The ideal scenario would be a technological-based platform providing a server storage facility, which would allow immediate cross-site access to confidential breast reconstruction patient information as soon as it becomes uploaded at one site. This would be used exclusively for reconstruction patients with appropriate EU General Data Protection and Regulation (GDPR). The creation of such a platform would be far more practical and cost-effective than generalized harmonization of information technology (IT) services across different NHS Hospital Trusts, which would involve great expense. This would allow immediate access to critical information for all necessary parties and ensure that as soon as it is available at one site it would be visible on multiple sites. Additionally, it avoids replication of workload, highlighted by the current scenario of multiple emails being received by the MDT co-ordinator—up to 11—for each patient. Clearly, this would require investment for such an IT infrastructure project. Unfortunately, within financially constrained health care services, the priority to adopt such an approach is unlikely to be high enough across multiple NHS Trusts. An alternative is to ensure that the oncological surgery is performed by a surgeon from the referring unit where all the cancer work-up has been performed, allowing unfettered access to all necessary information. In this evaluation, only one-third (9/31) of patients from the referral centres had their surgery performed by a surgeon who originally diagnosed their cancer. In terms of work-force planning and capacity, this is likely impractical unless further surgical staff are employed or allocated such a role as to specifically cover the reconstruction cases from their units. This would ensure correct surgery on the day of operation, but the patient information—possessed by the surgical oncologist from the remote site—would still need to be available post-operatively at the tertiary centre for adjuvant treatment management and should complications develop warranting intervention. Ultimately, this means that the information does need to be transferred in some form. The establishment of a formal proforma—demonstrating the critical information from the ABS/BAPRAS guidelines that requires transfer—for which, each item must be completed, and accompanying documents supplied before the patient can be seen in the tertiary centre may be a more practical immediate solution. This documentation should be vetted at the receiving unit by the oncoplastic fellow—managing the organization of the reconstruction lists—and on their satisfaction with the transferred data content, an outpatient clinic appointment arranged.

## Conclusions

Demand for breast reconstruction continues to increase and provision of this service is essential for cancer patients. However, its performance must not come at the expense of compromising the availability of critical oncoplastic information and patient safety through poor clinical governance. Without such issues being rectified, it is almost an inevitability that avoidable serious adverse events will occur at some stage, irrespective of the meticulous attention paid by the operating surgeon to compensate for the logistical shortcomings.

## Conflicts of interest statement

The authors have no disclosures to make concerning financial and personal relationships with other people or organisations that could inappropriately influence their work. No ethical approval was requested for this work.

## Funding

The authors did not receive any funding for this work.
